# Ethanol Extract of Chinese Propolis Facilitates Functional Recovery of Locomotor Activity after Spinal Cord Injury

**DOI:** 10.1155/2011/749627

**Published:** 2010-09-08

**Authors:** Masaki Kasai, Hidefumi Fukumitsu, Hitomi Soumiya, Shoei Furukawa

**Affiliations:** Laboratory of Molecular Biology, Gifu Pharmaceutical University, Daigaku-nishi 1-25-4, Gifu 501-1196, Japan

## Abstract

An ethanol extract of Chinese propolis (EECP) was given intraperitoneally to rats suffering from hemitransection of half of their spinal cord (left side) at the level of the 10th thoracic vertebra to examine the effects of the EECP on the functional recovery of locomotor activity and expression of mRNAs of inducible nitric oxide (NO) synthase (iNOS) and neurotrophic factors in the injury site. Daily administration of EECP after the spinal cord injury ameliorated the locomotor function, which effect was accompanied by a reduced lesion size. Furthermore, the EECP suppressed iNOS gene expression, thus reducing NO generation, and also increased the expression level of brain-derived neurotrophic factor and neurotrophin-3 mRNAs in the lesion site, suggesting that the EECP reduced the inflammatory and apoptotic circumstances through attenuation of iNOS mRNA expression and facilitation of mRNA expression of neurotrophins in the injured spinal cord. These results suggest that Chinese propolis may become a promising tool for wide use in the nervous system for reducing the secondary neuronal damage following primary physical injury.

## 1. Introduction

Propolis is a natural substance collected by honeybees from buds and exudates of certain trees and plants, and is used in the beehive as a protective barrier against enemies. Propolis, which has been used in folk medicines in many regions of the world for centuries [1], is reported to contain over 300 compounds including alcohols, aldehydes, aromatic acids and esters, flavonoids, fatty acids, terpenoids, steroids, and carbohydrates [2–4]. As some of these compounds exert antioxidant [5], antibacterial [6], antiviral [7], anti-inflammatory [8], and anticancer [9] activities, propolis is extensively used in foods and beverages to maintain health and to prevent or lessen the severity of diseases such as inflammation, heart disease, diabetes, and cancer [2, 10]. 

Bee products are useful tools to protect against and to provide therapy for some particular neurological disorders [11]. With regard to the effects of propolis on the nervous system, evidence showing that propolis and/or its components are neuroprotective against various brain insults *in vivo* or neuronal damage *in vitro* has been rapidly accumulating. For instance, caffeic acid phenethyl ester (CAPE, [12–14],) pinocembrin [15], and water-soluble components of propolis [16] are neuroprotective against excitotoxic insults in ischemia/reperfusion injury. Furthermore, water-soluble components of propolis can ameliorate scopolamine-induced learning and memory impairment in mice [17]; they play a protective role against oxidative stress-induced damage to the retina [18]. Although these protective activities may be partially due to radical-scavenging actions, the regulation of signal transduction, such as inhibition of NF*κ*B and activation of c-Jun N-terminal kinase, is also postulated to be involved [19]. Thus, many reports indicate that propolis and its constituents protect against neuronal death at least partly by the mediation of their antioxidant activity. However, little is known about the effects of propolis on neurite outgrowth or axonal regeneration except for a recent finding showing that one of the major components of Brazilian propolis, artepillin C, stimulates neurite outgrowth from cultured rat pheochromocytoma PC12 cells [20]. 

 That a regenerative response is critically limited in the central nervous system of mammals is a serious problem, although many experimental strategies have been employed to minimize tissue damage and to enhance axonal growth and regeneration. In the case of a spinal cord injury (SCI), the failure of axonal regeneration is thought to result partly from the expression of axonal growth-inhibiting molecules [21], the lack of neurotrophic factors [22], and/or inflammatory reactions [23]. Among these possibilities, inflammation is a response that occurs immediately after SCI, and is likely to cause secondary injury that magnifies the primary injury and facilitates neuronal dysfunction. In the CNS, nitric oxide (NO) plays important roles under normal circumstances and also seems to be involved in the pathophysiology observed under ischemic and traumatic conditions [24, 25]. NO is increased in the injured site after SCI; and the NO- induced might be neurotoxic [26], which is consistent with a finding that NO is closely involved in the development of pathological processes *in vivo* such as posttraumatic spinal cord cavitation [27]. 

Therefore, we evaluated the effect of an ethanol extract of Chinese propolis (EECP), because many biologically active constituents [28], including neuroprotective ones such as pinocembrin [29], are in abundance in the methanol or ethanol extract of this substance. EECP was given intraperitoneally to rats, and the expression of mRNAs of iNOS and neurotrophic factors in the injury site and the effect on functional recovery after SCI were examined. We found a novel conspicuous activity of EECP that suppressed iNOS gene expression, thus reducing NO generation, and increased the expression level of brain-derived neurotrophic factor (BDNF) and neurotrophin-3 (NT-3) mRNAs in the lesion site. In addition, EECP ameliorated the locomotor activity after SCI, which improvement was accompanied by a reduced lesion size in the injury site. 

These results suggest that Chinese propolis may become a promising tool for wide use in the nervous system for reducing the secondary neuronal damage following a primary physical injury.

## 2. Subjects and Methods

### 2.1. Animal Surgery

Female Wistar rats (7 weeks of age, weighing 120–140 g; Japan SLC, Hamamatsu, Japan) were used in this study, and were handled in accordance with the Guidelines of Experimental Animal Care issued by the Office of the Prime Minister of Japan. The animals were anesthetized with sodium pentobarbital (40 mg/kg body weight), and the half of the spinal cord (left side) was hemitransected by microsurgical scissors after laminectomy at the level of the 10th thoracic vertebra. After arrest of hemorrhage, the muscle and skin were sutured. The rats were then placed in normal cages and given free access to food and water.

### 2.2. Administration of EECP

 Chinese propolis was collected in the Shangdong Province of China from July to August. It was extracted with 95% ethanol while being stirred for 24 hours at room temperature to yield the EECP. The EECP thus prepared was a generous gift from Api Co., Ltd., Gifu, Japan. It was diluted with sterile phosphate-buffered saline (PBS) and intraperitoneally administered to the animals (0.2, 1 or 5 mg/kg) immediately after the injury and then once every 24 hours for 3 weeks. The vehicle-treated animals received PBS- containing ethanol whose final concentration was equal to that of the EECP solution.

### 2.3. Assessment of Locomotor Function

Locomotor function of the left hindlimb in which neural transmission had been halted ipsilaterally was assessed by the Basso, Beattie, and Bresnahan (BBB) locomotor scale in openfield as described earlier [30]. Evaluation was performed once a day for 3 weeks immediately after the SCI by observers blinded to the experimental treatments.

### 2.4. Reverse Transcription-Polymerase Chain Reaction (RT-PCR)

 For the RT-PCR experiment, the spinal cords of rats that had received daily administration of vehicle or EECP were dissected out immediately after the hemitransection or 1, 3, 7, or 14 days later; and the segments just rostral or caudal to the injury site (2-mm length for each) were collected. Total RNA was isolated from the collected spinal cords by using TRIZOL Reagent (Invitrogen) according to the manufacturer's instruction. All RNA samples were treated with DNase I to remove contaminating genomic DNA. RT-PCR was performed as described previously in [31] to assess the mRNA levels of iNOS, BDNF, NT-3, and glial cell line-derived neurotrophic factor (GDNF). *β*-Actin was used as the internal control. The amplification was carried out with a thermal cycler at 94°C for 5 minutes, followed by 24–38 cycles consisting of 94°C for 30 second, 60–63°C for 1 minute, and 72°C for 45 second. The sequences of primers, annealing temperature, and size of PCR product were as follow: for the *β*-actin forward primer, 5′-GCCGTCTTCCCCTCCATCGT-3′, and reverse primer, 5′-CCCGTCTCCGGAGTCCATCA-3′, at 63°C (390 bp); for the iNOS forward primer, 5′-GCTGGAGGTGACCATGGAGCAT-3′, and reverse primer, 5′-CCTGGCTAGCGCTTCCGACTTT-3′, at 63°C (688 bp); for the BDNF forward primer, 5′-CCCAGGGCAGGTTCGAGAGG-3′, and reverse primer, 5′-CCCGCCAGACATGTCCACTG-3′, at 61°C (350 bp); for the NT-3 forward primer, 5′-TTACCAGAGCACCCTGCCCAAA-3′, and reverse primer, 5′-ACCTGGTGTCCCCGAATGTCAA-3′, at 61°C (348 bp); and for the GDNF forward primer, 5′-GAGAGGAATCGGCAGGCTGCAGCTG-3′, and reverse primer, 5′-CAGATACATCCACATCGTTTAGCGG-3′, at 60°C (337 bp). After amplification, PCR products were subjected to 2% agarose gel electrophoresis and visualized by ethidium bromide staining. The images were captured with FLA-5100 (FUJIFILM, Tokyo, Japan). The optical density of each band was quantified by utilizing image-analysis software (NIH ImageJ).

### 2.5. Tissue Preparation for Immunohistochemical Analysis

The animals were deeply anesthetized by an intraperitoneal injection of sodium pentobarbital and then cardio-perfused with 4% paraformaldehyde (PFA) in 0.1 M phosphate buffer (pH 7.4). The spinal cord tissues including the lesion site were dissected out and postfixed in the same fixative overnight at 4°C. The tissues were then soaked in cold PBS containing 20% sucrose for 15 hours, and subsequently frozen in embedding compound (Sakura Finetek, Tokyo, Japan). Frozen sections (30-*μ*m thickness) were prepared with a cryostat (model CM 1800; Leica, Nussloch, Germany) and placed on adhesive-coated slides (Matsunami, Osaka, Japan).

### 2.6. Immunohistochemical Analysis

Cryostat sections were fixed with 4% PFA solution for 10 minutes at 37°C and rinsed for 30 minutes at 37°C in 0.1 M Tris-HCl buffer (pH 7.4) containing 0.3% (v/v) Triton X-100. After having been washed with PBS, the sections were blocked for 30 min at room temperature in PBS containing 2% Block Ace and then reacted with the diluted primary antibody specific for glial fibrillary acidic protein (GFAP; 1 : 1000, Dakocytomation, Glostrup, Denmark) for appropriate times at 4°C. After another wash with PBS, the sections were incubated with fluorescence-conjugated secondary antibody for 3 hours at room temperature (Alexa 546-conjugated goat anti-rabbit IgG; 1 : 1000, purchased from Invitrogen, Carlsbad, CA). The slides were washed with PBS and coverslipped with PermaFluor Aqueous Mounting Medium (Thermoshandon, Waltham, CA). Finally, the images were observed with a confocal laser microscope (LSM 510; Zeiss).

### 2.7. Image Analysis

 Lesion size of the injury site was measured by tracing the lesion area within the borders of the lesions as defined by GFAP labeling through 5 representative horizontal sections, the positions being 0.12 and 0.24 mm ventral to the midline, at the midline, and 0.12 and 0.24 mm dorsal to the midline in each rat.

### 2.8. Statistical Analysis

 All numerical data were presented as group mean values with standard error (SE). Statistical analysis of locomotor function assessed by using the BBB scale and that of various gene expressions in the injured spinal cord were performed by two-way analysis of variance (ANOVA) followed by the Bonferroni posttest. A statistically significant difference in the size of the lesion site was determined by performing Student's *t*-test.

## 3. Results

### 3.1. Locomotor Recovery of SCI Rats with EECP Treatment

**Figure 1:**
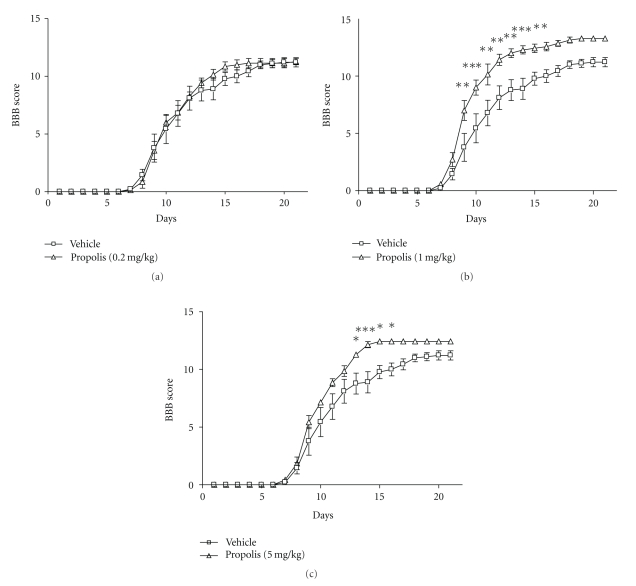
EECP accelerated locomotor functional recovery after hemi-transection of the rat spinal cord. Changes in the locomotor function of the vehicle-treated (*n* = 9) and EECP-treated animals (*n* = 7) during the 3-week experimental period are shown. EECP-treated group (1 mg/kg, (b) 5 mg/kg, (c)) showed a significant behavioral improvement compared with the vehicle-treated group. However, a low dose of EECP (0.2 mg/kg, (a)) had no effect on the recovery of locomotor function after SCI. Significance of differences from vehicle-treated group was determined by using repeated measures of two-way ANOVA followed by the Bonferroni post-test. *P < .05, **P < .01, and ***P < .001 versus vehicle-treated group.

 We evaluated the effect of various doses of EECP on the locomotor activity of the left hindlimb of hemi-transected rats for over 3 weeks after the SCI by using the BBB rating scale. Hemi-transection of the spinal cord resulted in complete paralysis of the hindlimb ipsilateral to the transection side (left) for several days in all groups. Afterwards, the locomotor function was restored rapidly, and attained a plateau value (11-12 points) of the BBB rating scale by 3 weeks after the injury in the vehicle-treated group (Figure 1). The animals that had received a low dose of EECP (0.2 mg/kg) did not show any additional improvement throughout the test period (Figure 1(a)) whereas significantly more rapid and higher improvement of locomotor function was observed in the rats that had received an intraperitoneal administration of 1 or 5 mg/kg of EECP (Figures 1(b) and 1(c)). Particularly, the largest restoration occurred in the animals that had received 1 mg/kg of EECP, for which the value of the BBB rating scale was significantly higher than that of the vehicle-treated group between experimental days 9 and 16.

### 3.2. Evaluation of mRNA Levels of iNOS and Neurotrophic Factors in the Site of SCI

**Figure 2:**
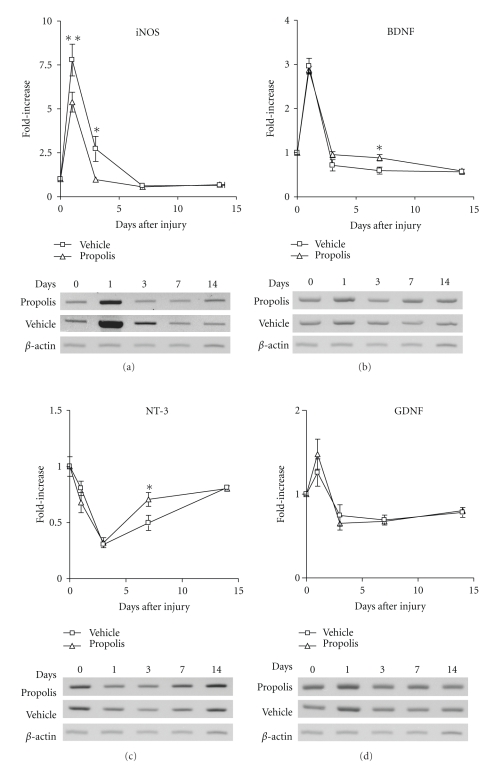
EECP attenuated the expression of iNOS mRNA and enhanced that of BDNF and NT-3 mRNAs in the site of SCI. Vehicle or EECP (1 mg/kg) was daily administered to rats with SCI. The results of RT-PCR analysis of various genes in the injured spinal cord of rats that had received daily administration of vehicle or EECP are shown.** (**a) The expression level of iNOS mRNA in the injured spinal cord was significantly lower in the EECP-treated group (*n* = 3) than in the vehicle-treated group (*n* = 3) on experimental days 1 and 3. (b), (c) and (d) mRNA expression levels of BDNF (b) and NT-3 (c) were significantly higher in the EECP-treated group (*n* = 6) than in the vehicle-treated group (*n* = 6) on experimental day 7, but there was no significant difference in the GDNF (d) mRNA level between the 2 groups (*n* = 3). Significance of differences from vehicle-treated group was determined by using two-way ANOVA followed by the Bonferroni post-test. *P < .05, **P < .01 versus vehicle-treated group. *β*-Actin was used as the internal control.

 To assess the anti-inflammatory effect of EECP on the injured spinal cord, we first examined by RT-PCR the expression level of iNOS mRNA in the injury site in rats that had received daily administration of vehicle or EECP (1 mg/kg). The expression of iNOS mRNA markedly increased and attained its maximal level 1 day after SCI, and gradually decreased to the original level irrespective of EECP administration. The expression level of iNOS mRNA was significantly lower in the EECP-treated group than in the vehicle-treated group, when evaluated 1 day or 3 days after the SCI and subsequent EECP administration (Figure 2(a)). 

Next, the mRNA levels of neurotrophic factors BDNF, NT-3, and GDNF in the site of SCI were examined. Expression of BDNF and GDNF mRNAs was evoked in response to the injury, attained its maximal level on experimental day 1, and rapidly decreased on experimental day 3 in both vehicle- and EECP-treated groups (1 mg/kg). Furthermore, the BDNF mRNA of the vehicle-treated group decreased to a level lower than that of the EECP-treated group on experimental day 7, implying that EECP sustained the BDNF mRNA level significantly higher, which level would have otherwise decreased at that time (Figure 2(b)). In contrast, the expression of NT-3 mRNA was rather decreased in response to the injury, attained its minimal level on experimental day 3, and gradually recovered to the original level in both groups. EECP limitedly but significantly enhanced the recovery of the decreased expression of NT-3 mRNA on experimental day 7 (Figure 2(c)). EECP did not affect the expression of GDNF mRNA in the lesion site throughout the experimental period (Figure 2(d)).

### 3.3. Decrease in Size of Spinal Cord Lesion after EECP Administration

**Figure 3:**
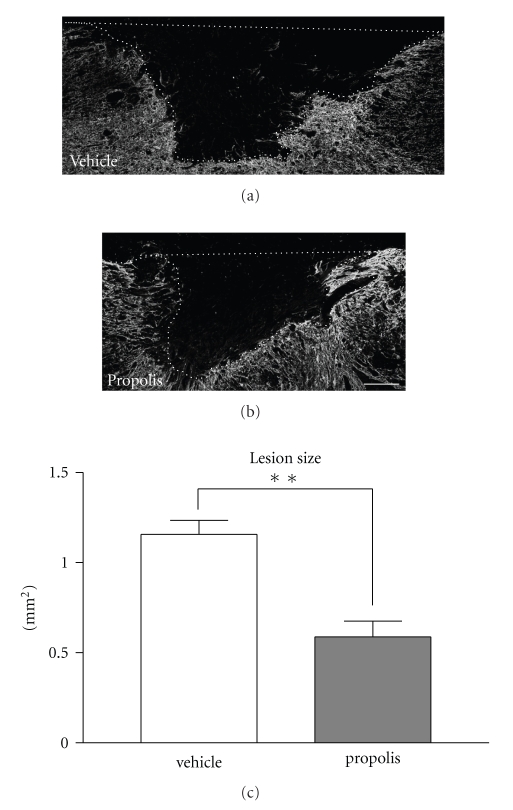
EECP reduced the lesion size in the injured spinal cord. Vehicle or EECP (1 mg/kg) was daily administered to rats with SCI. Fluorescence photographs of immunoreactivity of GFAP in the SCI lesion site of the vehicle-treated group (*n* = 3) (a)) and in that of the EECP-treated group (*n* = 3) (b)) are shown. The area surrounded by the dashed line (lesion size, (a), (b)) was compared between the EECP-treated and the vehicle-treated groups (c). The lesion size of the EECP-treated group was significantly smaller than that of the vehicle-treated group on experimental day 14. The left side is rostral. Significance of differences from vehicle-treated group was determined by using Student's *t*-test. **P < .01 versus vehicle-treated group. Scale bar: (a), (b), 200 *μ*m.

 To assess histologically the anti-inflammatory effect of EECP on the injured spinal cord, we measured on experimental day 14 the size of the lesion site in the spinal cord of rats that had received daily administration of vehicle or EECP (1 mg/kg). The EECP-treated animals had significantly smaller lesions than the vehicle-treated ones (Figure 3).

## 4. Discussion

 In this study, we found that EECP (1) accelerated repair of the SCI-induced locomotor function, (2) decreased the level of iNOS mRNA in the lesion site of the spinal cord and increased that of BDNF and NT-3 mRNAs, and (3) decreased the lesion size in the injured spinal cord of the rats. 

In the CNS, NO is involved in a variety of biological processes to maintain homeostasis; however, it also plays a detrimental role as a potent oxidant during pathophysiological processes occurring in neural tissues [25]. Under pathological circumstances, excessive NO is mainly produced by injury-activated iNOS, and causes apoptosis [32]. Therefore, the strategy to reduce iNOS expression or suppress NO production in the lesion site can be a practical approach to prevent or repair the injury-induced neuropathological dysfunction. It is known that an ethanol extract of propolis inhibits the gene expression and enzyme activity of iNOS [33], which is at least partly responsible for the neuroprotective activity afforded by the components of propolis [13, 15]. However, the effect of the EECP on traumatic SCI remained to be elucidated. Our present study clearly demonstrated that the EECP attenuated the expression of iNOS mRNA and reduced the lesion size in the injured spinal cord (Figures 2 and 3), consistent with our previous finding that pyrroloquinoline quinine, a naturally occurring redox cofactor, improves locomotor function after SCI by lowering iNOS gene expression [34]. It was earlier reported that injury-induced NO is involved in the formation of the cavity after damage to the spinal cord, which formation is followed by loss of neurological function [26], suggesting that the EECP lightened the secondary damage after SCI via attenuation of iNOS gene expression. We consider the cells predominantly expressing injury-evoked iNOS mRNA to have been inflammatory cells such as macrophages invading the damaged tissue; because the iNOS-expressing cells that invaded the lesioned area were positive for ED-2, a marker of macrophages and/or perivascular cells, and their population peaked at 1 day and then declined, disappearing 3 days after the injury [32], consistent with the time course of iNOS mRNA expression in the injury site (Figure 2). 

Our present study revealed that the EECP could modify upwardly the injury-evoked mRNA expression of BDNF and NT-3 on experimental day 7 (Figure 2). We propose that upregulation of these neurotrophins contributed partly to the restoration of the functional locomotor activity, because neurotrophins such as BDNF and NT-3 delivered to the injury site induce axonal sprouting and/or axonal regeneration followed by improvement of locomotor activity after SCI [35, 36]. As another profitable effect of the EECP on neuronal function, our preliminary results (Kasai et al., unpublished data) suggest that the EECP activates the mitogen-activated protein kinase (MAPK)/extracellular signal-regulated kinase (ERK) 1/2 signaling pathway (ERK1/2) in cultured cortical neurons. The ERK1/2 signaling pathway is preferentially activated in response to extracellular stimuli, including growth factors and neurotrophic factors [37]. For instance, BDNF and NT-3 bind to high-affinity tyrosine kinase receptors, TrkB and TrkC, respectively, and activate the ERK1/2 signaling pathway to influence growth, development, differentiation, and survival of neurons [38]. Therefore, the EECP may mimic at least partly the signaling pathway of BDNF and/or NT-3, facilitating axonal regeneration in the injured spinal cord. The EECP may thus facilitate improvement of hindlimb locomotor function after SCI.

 What components in the EECP are responsible for the beneficial effect found in present study? Numerous studies, carried out with the combined efforts of phytochemists and pharmacologists, have led in recent years to the idea that different propolis samples could be completely different in their chemistry and biological activity [4]. It has been reported that Chinese propolis contains many biologically active constituents expected to be useful for improvement of the neuropathological conditions in the injured spinal cord [28]. For example, CAPE is a constituent identified to exert antiinflammatory activity through inhibiting the gene expression and catalytic activity of iNOS [39], and to protect the brain from ischemia-reperfusion injury [13]. Therefore, CAPE is one of the most probable candidates that may act beneficially after traumatic SCI. Chrysin, the flavonoid of highest concentration in Chinese propolis, potentially represents anti-oxidative capacity in neuronal cell death and exhibits an anti-inflammatory effect by inhibiting iNOS mRNA and NO production [40, 41]. Additionally, pinocembrin, also one of the main flavonoids in Chinese propolis, exerts its neuroprotective effect on oxygen-glucose deprivation/reoxygenation-injured cortical neurons based on a probable mechanism involving a reduction in iNOS [15]. It is highly possible that these flavonoids can reduce the inflammatory and/or apoptotic circumstance induced after SCI. Artepillin C, though a major component of Brazilian propolis, induces neurite outgrowth from PC12m3 cells, a cell line established from parental PC12 cells, via ERK1/2 and p38 MAPK pathways [20]. Thus, artepillin C might be a candidate to stimulate axonal regeneration in the injured spinal cord. It is likely that the neurological outcomes observed in our present study could have resulted from the biological activities of plural constituents of propolis. Evaluation of the effective constituent(s) on locomotor functional recovery is now in progress in our laboratory. Furthermore, we are planning to search for unknown active components that possess the potential to repair the SCI. For overcoming the critical issue of variation in the quality of propolis preparations, the biological properties of EECP from preparations of different chemical composition must be compared.

**Figure 4:**
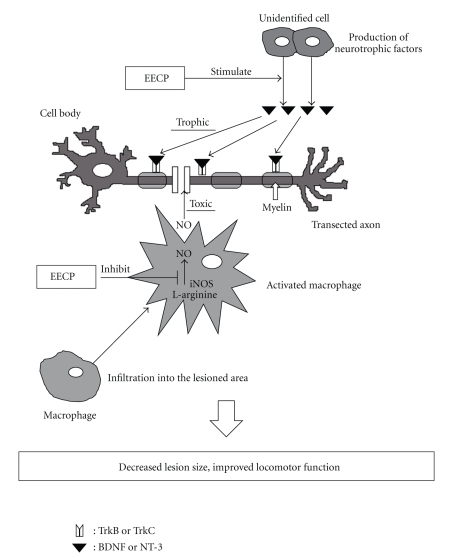
Hypothetical diagram showing mechanism of the effect of EECP on spinal cord neurotrauma. EECP attenuates the expression iNOS mRNA, probably derived from macrophages that have infiltrated into the lesioned area. Furthermore, EECP increases BDNF and NT-3 gene expression in the injured spinal cord, which may protect neurons and myelin (elaborated by oligodendrocytes) and/or cause regeneration of the transected axons. These effects of EECP may partially contribute to the decreased lesion area and improved locomotor function after SCI.

 In summary, we demonstrated that the intraperitoneal administration of EECP reduced the inflammation and/or apoptosis through attenuating iNOS mRNA expression, but increased BDNF and NT-3 mRNA expression, in the injured spinal cord. These changes resulted in the reduced lesion size and improved locomotor function after SCI (Figure 4). Although identification of the active constituents of the EECP, elucidation of the critical action mechanisms, and evaluation to decide the validity of administering EECP by oral administration still remain to be resolved, propolis and its active constituents may be developed in the future as evidence-based complementary and alternative medicines and be expected as promising tools for the treatment of SCI.
